# Quantitative Analysis of Major Constituents in Green Tea with Different Plucking Periods and Their Antioxidant Activity

**DOI:** 10.3390/molecules19079173

**Published:** 2014-07-01

**Authors:** Lan-Sook Lee, Sang-Hee Kim, Young-Boong Kim, Young-Chan Kim

**Affiliations:** Korea Food Research Institute, Seongnam, Kyonggi 463-746, Korea; E-Mails: sohee0809@hanmail.net (L.-S.L.); kimsh@kfri.re.kr (S.-H.K); kybaaa@kfri.re.kr (Y.-B.K)

**Keywords:** green tea, plucking period, antioxidant activity, ABTS, FRAP, DPPH

## Abstract

The objective of this study was to determine the relationship between the plucking periods and the major constituents and the antioxidant activity in green tea. Green tea was prepared from leaves plucked from the end of April 2013 to the end of May 2013 at intervals of one week or longer. The contents of theanine, theobromine, caffeine, catechin (C), and gallocatechin gallate (GCg) were significantly decreased, whereas those of epicatechin (EC), epigallocatechin gallate (EGCg) and epigallocatechin (EGC) were significantly increased along with the period of tea leaf plucking. In addition, antioxidant activity of green tea and standard catechins was investigated using ABTS, FRAP and DPPH assays. The highest antioxidant activity was observed in relatively the oldest leaf, regardless of the assay methods used. Additionally, the order of antioxidant activity of standard catechins was as follows: EGCg ≥ GCg ≥ ECg > EGC ≥ GC ≥ EC ≥ C. Moreover, the *cis*-catechins contents were the key factor affecting the antioxidant activity of green tea in all assays employed (ABTS, r = 0.731, *p* < 0.01; FRAP, r = 0.886, *p* < 0.01; DPPH, r = 0.778, *p* < 0.01).

## 1. Introduction

Tea, derived from leaves of the plant *Camellia sinensis*, is the most widely consumed beverage in the world and can be categorized into three main types depending on the level of oxidation: green (unfermented), oolong (partially fermented) and black (fermented) tea [1]. The chemical composition of green tea varies with genetic strain, climatic conditions, soil properties, plucking season, position of the leaf, processing and storage [2,3,4]. Some factors are more important than others; for example, the highest quality green teas are plucked during the first flush in late April and early May and quality declines in later harvests [5]. Usually, the buds and the first two to three leaves are plucked by hand or a mechanical tea plucker for processing. This process is generally repeated every one to two weeks. These basic types of tea have different quality characteristics, including appearance, flavor, taste, and color [6].

The relationship between the quality and chemical components in green tea have been studied, and have shown that free amino acids, caffeine and polyphenols are qualitatively important components. Especially, catechins, the main component of polyphenols, are well known for their antioxidant properties, which have led to their evaluation in many diseases associated with free radicals, including cancer, cardiovascular and neurodegenerative diseases [7,8,9,10,11,12].

Generally, the major catechins of tea leaves are (+)-catechin (C), (−)-epicatechin (EC), (+)-gallocatechin (GC), (−)-epigallocatechin (EGC), (−)-epicatechin gallate (ECg), (−)-epigallocatechin gallate (EGCg), and (+)-gallocatechin gallate (GCg) [1]. The antioxidant properties of catechins are mainly related to the number and position of hydroxyl group in the molecules and consequently binding and neutralization of free radicals by these hydroxyl groups [13,14,15]. Previous studied have shown that tea catechins are excellent electron donors and effective scavengers of physiologically relevant reactive oxygen species *in vitro*, including superoxide anions [15,16,17,18], peroxyl radicals, and singlet oxygen [15]. Most studies on the antioxidant effects of green tea are directly related to the total phenolic extracts, without considering the contributions of individual molecules, although various catechins, such as EGCg, ECg and EGC, have been linked to strong antioxidant activity in green tea extracts.

Therefore, in this study, the chemical compositions and antioxidant activities of green tea with different plucking periods were determined and the resultant data were applied to correlation analysis to find important factors for contributing to the antioxidant activity. Additionally, we have re-examined using individual catechins standards with Trolox to verify the apparent relationship between individual catechins and antioxidant activity. Considering the cost and health benefit, it is important to evaluate the antioxidant activities of commonly consumed green teas, because the price of commercially available green tea is determined by the quality of tea leaf. ABTS, DPPH and FRAP assays were used to measure the antioxidant activity.

## 2. Results and Discussion

### 2.1. Identification and Quantification of Major Constituents in Green Tea

**Figure 1:**
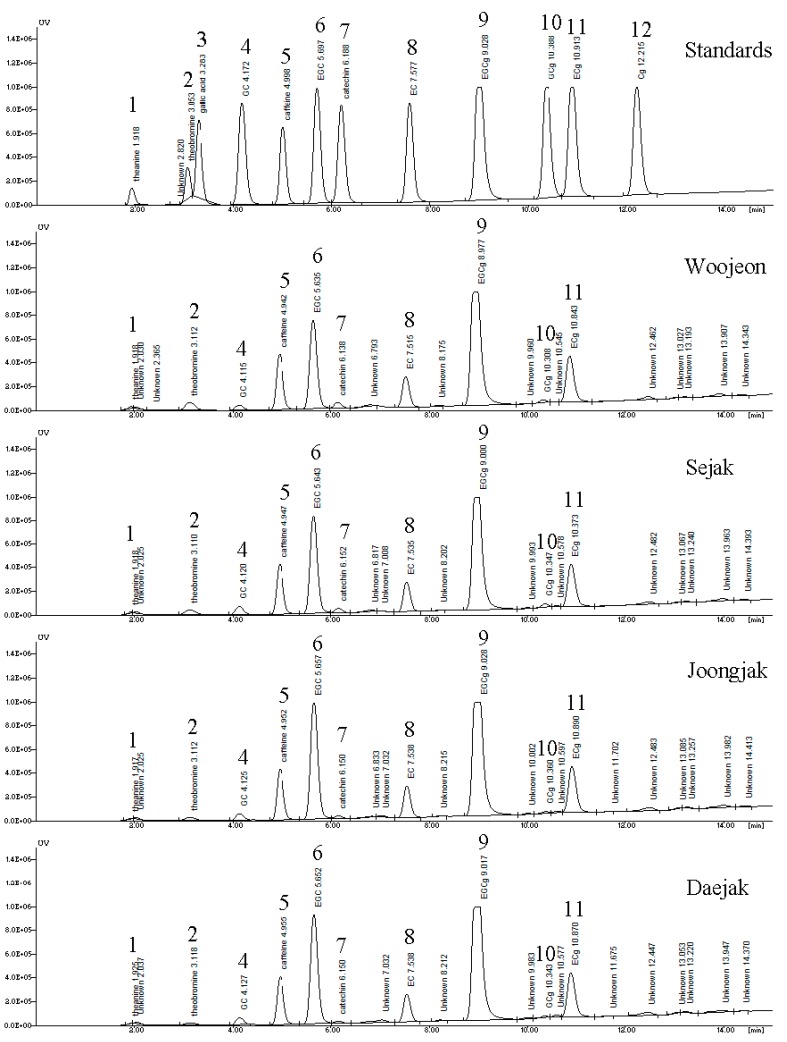
HPLC chromatograms of standard solution and green tea extracts with different plucking periods. Peaks correspond to (**1**) theanine, (**2**) theobromine, (**3**) gallic acid, (**4**) (+)-gallocatechin, (**5**) caffeine, (**6**) (−)-epigallocatechin, (**7**) (+)-catechin, (**8**) (−)-epicatechin, (**9**) (−)-epigallocatechin gallate, (**10**) (+)-gallocatechin gallate, (**11**), (−)-epicatechin gallate and (**12**) (+)-catechin gallate. Woojeon, first flush leaf of late April; Sejak, second flush leaf of early May; Joongjak, third flush leaf of mid-May; Daejak, fourth flush leaf of late May.

Ten major compounds were identified and quantified from green tea with different plucking periods, as shown in Figure 1 and Table 1. Peak compound identification in green tea extracts was achieved by comparing HPLC retention times and PDA-UV spectra in chromatograms of standards with those found in the chromatograms of prepared extracts and quantification was achieved through calibration curve with external standards by HPLC (Figure 1).

From the data presented in Table 1, the contents of theanine, theobromine, caffeine, catechin, and GCg were found to be significantly decreased along with the period of tea leaf plucking. In contrast, the contents of EC, EGCg and EGC were significantly increased along with the period of tea leaf plucking. However, among catechins, GC and ECg did not tend to constant for content. Moreover, we have calculated gallated catechins, non-gallated catechins and total catechins from quantification value of individual catechins. The gallated catechins are derived as the sum of EGCg, GCg and ECg. The non-gallated catechins are derived as the sum of GC, EGC, C and EC. The total catechins are derived as the sum of GC, EGC, C, EC, EGCg, GCg and ECg. These values were found to be very influenced by the components making up that parameter, and especially EGCg and ECg have an important role in the content of parameters. In other words, non-gallated and total catechins were significantly increased along with the period of tea leaf plucking, but gallated catechins did not tend to constant for content.

**Table 1:** Variation in content of theanine, theobromine, caffeine and catechins in green tea with different plucking periods.

Tea Compound	Content (mg/g Green Tea)			
	Woojeon	Sejak	Joongjak	Daejak
Theanine	6.46 ± 0.83 ^a^	4.60 ± 0.90 ^b^	2.84 ± 0.11 ^c^	2.19 ± 0.68 ^c^
Theobromine	8.81 ± 0.19 ^a^	6.25 ± 0.03 ^c^	3.89 ± 0.11 ^c^	2.83 ± 0.05 ^d^
Caffeine	29.54 ± 0.28 ^a^	27.21 ± 0.50 ^b^	27.19 ± 0.25 ^b^	26.74 ± 0.55 ^b^
GC	2.28 ± 0.06 ^d^	3.65 ± 0.13 ^a^	2.96 ± 0.08 ^c^	3.19 ± 0.11 ^b^
EGC	30.52 ± 0.32 ^d^	34.75 ± 0.72 ^c^	38.86 ± 0.75 ^b^	40.34 ± 0.50 ^a^
C	2.48 ± 0.03 ^a^	1.89 ± 0.03 ^b^	1.20 ± 0.02 ^c^	0.99 ± 0.02 ^d^
EC	11.84 ± 0.11 ^b ^	11.86 ± 0.25 ^b^	12.00 ± 0.18 ^b^	12.59 ± 0.21 ^a^
EGCg	105.37 ± 0.71 ^b^	103.95 ± 1.32 ^b^	111.59 ± 0.68 ^a^	112.86 ± 1.25 ^a^
GCg	6.73 ± 0.06 ^a^	6.76 ± 0.05 ^a^	5.70 ± 0.04 ^b^	5.61 ± 0.03 ^b^
ECg	41.19 ± 0.61 ^a^	38.14 ± 0.53 ^c^	39.89 ± 0.35 ^b^	39.61 ± 0.65 ^b^
Gallated catechins	153.28 ± 1.32 ^b^	148.85 ± 1.87 ^c^	157.18 ± 0.94 ^a^	158.07 ± 1.91 ^a^
Non-gallated catechins	47.12 ± 0.44 ^d^	52.15 ± 1.12 ^c^	55.02 ± 0.79 ^b^	57.11 ± 0.57 ^a^
Total catechins	200.40 ± 1.71 ^b^	201.00 ± 2.92 ^b^	212.20 ± 1.51 ^a^	215.19 ± 2.20 ^a^

All values are mean ± SD. Values within a row with different letters are significantly different by ANOVA with Tukey’s *post hoc* test at *p* < 0.05. GC, (+)-gallocatechin; EGC, (−)-epigallocatechin; C, (+)-catechin; EC, (−)-epicatechin; EGCg, (−)-epigallocatechin gallate; GCg, (+)-gallocatechin gallate; ECg, (−)-epicatechin gallate; Gallated catechins, sum of EGCg, GCg and ECg; Non-gallated catechins, sum of GC, EGC, C and EC; Total catechins, sum of GC, EGC, C, EC, EGCg, GCg and ECg. Woojeon, first flush leaf of late April; Sejak, second flush leaf of early May; Joongjak, third flush leaf of mid-May; Daejak, fourth flush leaf of late May.

There are different reports of major compound changes in green tea depending on the leaf age. Chen *et al.* reported that young tea leaves were richer in caffeine, EGCg and ECg than were mature leaves, but old leaves had higher levels of EGC and EC [19]. Lin *et al.* reported that young leaves were higher in caffeine, EGCg, EGC, ECg, EC, and catechin than were old leaves [20]. Lin *et al.* and Lee *et al.* observed that old leaves contained less caffeine, but more EGCg and EC than did young leaves, showing a good agreement with the results in the present study [3,21]. A possible reason for this discrepancy could be related to the plucking positions and seasons of tea leave collection. Chen *et al.* have compared the contents of individual catechins and caffeine among the tea leaves, which were plucked individually from the first (the youngest) to tenth (the oldest) ones in summer (August 2001) [19], whereas Lee *et al.* were plucked individually from the first (the youngest) to fourth (the oldest) ones in between spring and summer (June 2010) [21]. Additionally, Lin *et al.* have compared the catechins and caffeine contents between the old leaves in summer (August 1994) mixed with the tenth to fifth leaves and the youngest leaves (April 1995) mixed with the apical bud and the two youngest leaves [20]. However, we have compared the major constituents such as theanine, caffeine, theobromine and catechins among the tea leaves, which were plucked from the end of April to the end of May at intervals of one week or longer. Therefore, both different plucking positions and periods of tea leaves may cause different results on the composition of major components in green tea.

Generally, the price and quality of green tea decline with later plucking. Tea experts suggest that high quality green tea is made from the bud and the next two leaves, and sometimes the bud alone. Many studies have demonstrated the relationship between the green tea quality and the contents of caffeine and theanine in green tea, and their high contents are responsible for high quality of tea [1,22,23,24,25]. Furthermore, *cis*-catechins such as EGCg, EGC and ECg were identified as significant markers to determine the quality of the green tea [26,27,28]. Although, it has not yet been established to what extent tea quality is different according to the specific catechins content, most of these reports indicated that lower levels of EGC were associated with good quality of tea.

These findings have also been reported in the studies for assessing green tea quality through metabolite profiling of commercial green tea using NMR spectroscopy [5,28]. The authors of these reports showed that high quality green teas showed higher levels of caffeine, theanine, EGCg and ECg and lower levels of EGC and EC as compared to the low quality teas. These results are also consistent with our present study, although the analysis instrument used was different.

### 2.2. Total Antioxidant Activity in Green Tea Extracts and Standard Catechins

The results obtained for antioxidant activity of green tea by the ABTS, FRAP and DPPH assays are presented in Table 2. The three assays give very different values in absolute terms (ie, Trolox equivalents mM per g of green tea), but show the same relative pattern. Among samples, the highest antioxidant activity was observed in Daejak (relatively the oldest leaf) regardless of the assay methods used. This result agreement with Song *et al.*, who reported that the antioxidant activity of old leaf was higher than that in young leaf of green tea grown in Hawaii [29].

**Table 2:** Antioxidant activity of green tea with different plucking periods as determined by the ABTS, FRAP and DPPH assays.

Green Tea	Antioxidant Activity (mM TE/g Green tea)		
	ABTS	FRAP	DPPH
Woojeon	4,293.33 ± 216.05 ^NS^	1,555.06 ± 20.57 ^b^	1,423.22 ± 35.28 ^b^
Sejak	4,382.22 ± 262.77	1,568.73 ± 32.14 ^b^	1,441.00 ± 16.52 ^b^
Joongjak	4,687.78 ± 156.86	1,626.91 ± 1.29 ^ab^	1,478.78 ± 51.55 ^ab^
Daejak	4,682.22 ± 138.82	1,697.81 ± 6.37 ^a^	1,571.01 ± 58.09 ^a^

All values are mean ± SD. Values within a column with different letters are significantly different by ANOVA with Tukey’s *post hoc* test at *p* < 0.05. ^NS^, not significant. Woojeon, first flush leaf of late April; Sejak, second flush leaf of early May; Joongjak, third flush leaf of mid-May; Daejak, fourth flush leaf of late May.

In general, polyphenols have been thought to be responsible for most of the antioxidant activity in plant products. Green tea contains various classes of polyphenols, and up to 90% of its polyphenols are flavan-3-ols (catechins). Generally, tea catechin molecules have two benzene rings (commonly called the A- and B-ring) linked through a heterocyclic pyran or pyrone ring (c-ring) in the middle [30]. The catechin molecule also can exist as two geometrical isomers, *trans*-catechins and *cis*-epicatechins, depending on the stereochemical configuration of the 3',4'-dihydroxyphenyl and hydroxyl groups at the C-2 and C-3 of the C- ring. All catechins have hydroxyl groups at the C-5 and C-7 positions of A-ring. In contrast, the B-ring usually possessed two (EC, C, and ECg) or three (GC, EGC, GCg and EGCg) vicinal hydroxyl groups at C-3' and C-4', and C-5'. In some catechins, the C-3 position of the C-ring is linked with a gallate group through an ester bond ECg, GCg and EGCg. Many studies have reported that the scavenging effects of galloylated catechins (EGCg, ECg and GCg) were stronger than those of nongalloylated catechins (EGC, GC, EC and C), and the scavenging effects of EGC and GC, which have *ortho*-trihydroxyl groups in the B ring, were stronger than those of EC and C, which have *ortho*-dihydroxyl groups in the B ring [15,31,32,33,34]. Thus, it is suggested that the presence of the gallate group at the C-3 of the C-ring plays the most critical role in their free radical-scavenging abilities and an additional addition of the hydroxyl group at the C-5' of the B-ring also contributes to their scavenging activities.

**Figure 2:**
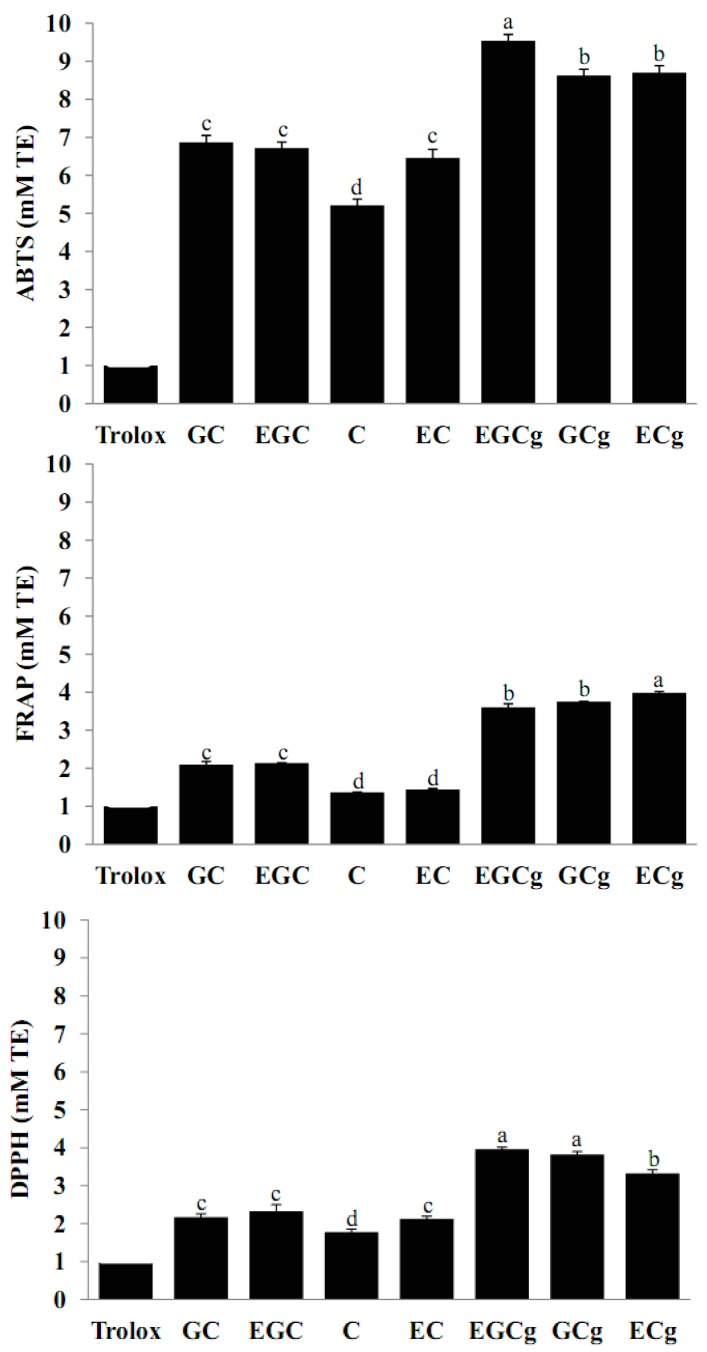
Antioxidant activity expressed as Trolox equivalent (mM TE) in standard catechins measured by ABTS, FRAP and DPPH assays. All values are mean ± SD. Values with different letters are significantly different by ANOVA with Tukey’s *post hoc* test at *p* < 0.05. GC, (+)-gallocatechin; EGC, (−)-epigallocatechin; C, (+)-catechin; EC, (−)-epicatechin; EGCg, (−)-epigallocatechin gallate; GCg, (+)-gallocatechin gallate; ECg, (−)-epicatechin gallate.

In the present study, these results were re-examined by ABTS, FRAP and DPPH assays using individual catechin standards with Trolox to verify the apparent relationship between individual catechins and antioxidant activity. This is needed because the comparison of antioxidant activity of individual catechins has not been performed by using the above three methods at the same time. As shown in Figure 2, the greatest antioxidant activity was observed in EGCg with the highest number of phenolic hydroxyl groups (eight groups), consistent with previous reports. Additionally, the antioxidant activities of EGCg, EGC and EC were same or higher than those of their corresponding epimers, respectively. However, this result is not consistent with previous study reported by Guo *et al.*, where they reported that the *trans*-catechins had higher antioxidant activities than the corresponding *cis*-epicatechins [15]. This disagreement is probably due to the different assay conditions used, particularly the concentration of standard catechins. The results also show that all catechins proved to be powerful antioxidants when compared to the Trolox positive control, which could achieve high antioxidant activity at a very low concentration. In this study, the order of antioxidant activity of standard catechins was as follows: EGCg (eight groups) ≥ GCg (eight groups) ≥ ECg (seven groups) > EGC (five groups) ≥ GC (five groups) ≥ EC (four groups) ≥ C (four groups). The results show that, the antioxidant activity of catechins depends largely on the number and position of hydroxyl groups in the molecule. In addition, they have metal chelation potential.

Moreover, the antioxidant activity has often been correlated with the phenolic content [35,36,37]. The strong antioxidant activities of green tea could be largely attributed to the high contents of EGCg, ECg and EGC since the these catechins represent about 80% of the total catechins in green tea. This result supports that antioxidant activity relates not only to the amounts of antioxidants, but also to the properties of antioxidants such as chemical structure and interactions among each other [38]. It is notable that the contents of individual catechins in green tea are affected by agronomic conditions such as plucking time and leaf age. It is expected that the results obtained in the present study will provide a broader understanding of bioactive compounds of green tea, and enable more informed decisions to be made in the plucking polices of tea leaves.

### 2.3. Relation between Antioxidant Activity and Catechin Composition

A Pearson correlation was used to assess the relationship between antioxidant activity and catechins composition and to establish their relative importance for antioxidant activity. Table 3 shows that the antioxidant activity had a positive and significant correlation with EGC and EGCg, but a negative and significant correlation with C and GCg by all assays. Additionally, the antioxidant activity was positively and significantly correlated with EC in FRAP and DPPH assays. However, the antioxidant activity was not correlated significantly with GC and ECg in all assays. These results confirm our above discussed results that the contents and structures of catechins greatly influence antioxidant activity in green tea. The high EGCg and EGC and low levels of C and GCg are therefore key factors determining antioxidant activity potential of green tea.

Moreover, in this study, various useful parameters are derived to determine the relationship between antioxidant activity and catechins composition, including, as mentioned above, total catechins, gallated catechins and non-gallated catechins. These parameters such as *trans*-catechins and *cis*-catechins are calculated from quantification value of individual catechins obtained by HPLC analysis. The *trans*-catechins are defined as the sum of GC, C and GCg. The *cis*-catechins are the sum of EGC, EC, EGCg and ECg. As the Table 3 showed, the antioxidant activity was positively and significantly correlated with total catechins, non-gallated catechins, *cis*-catechins and the ratio of *cis*/*trans* catechins, but negatively significant with *trans*-catechins. Among the factors affecting antioxidant activity, the *cis*-catechin had the highest correlations to all assays employed in this study (ABTS, r = 0.731, *p* < 0.01; FRAP, r = 0.886, *p* < 0.01; DPPH, r = 0.778, *p* < 0.01). All of the above results have shown that the antioxidant activity of green teas examined correlates well with the levels of individual catechins except for GC and ECg by ABTS, FRAP and DPPH assays. That was, the observed antioxidant activity of green teas with different plucking periods (various leaf ages) was statistically significant. This finding showed that antioxidant activity was increased with increasing leaf age.

**Table 3:** Correlations between antioxidant activity and catechins composition of green tea with different plucking periods.

Catechins	Pearson’s Correlation Coefficient ( r )		
	ABTS	FRAP	DPPH
GC	0.256	0.224	0.280
EGC	0.719 ^**^	0.868 ^**^	0.759 ^**^
C	−0.706 ^*^	−0.892 ^**^	−0.730 ^**^
EC	0.569	0.733 ^**^	0.727 ^**^
EGCg	0.710 ^**^	0.855 ^**^	0.748 ^**^
GCg	−0.684 ^*^	−0.891 ^**^	−0.693 ^*^
ECg	−0.133	−0.143	−0.132
Total catechins	0.741 ^**^	0.884 ^**^	0.795 ^**^
Gallated catechins	0.564	0.676 ^*^	0.601 ^*^
Non-gallated catechins	0.712 ^**^	0.846 ^**^	0.768 ^**^
Gallated/non-gallated ratio	−0.566	−0.659 ^*^	−0.600 ^*^
*cis*-Catechins	0.731 ^**^	0.886 ^**^	0.778 ^**^
*trans*-Catechins	−0.612 ^*^	−0.833 ^**^	−0.619 ^*^
*cis/trans* ratio	0.665 ^*^	0.870 ^**^	0.684 ^*^

GC, (+)-gallocatechin; EGC, (−)-epigallocatechin; C, (+)-catechin; EC, (−)-epicatechin; EGCg, (−)-epigallocatechin gallate; GCg, (+)-gallocatechin gallate; ECg, (−)-epicatechin gallate; Total catechins, sum of GC, EGC, C, EC, EGCg, GCg and ECg; Gallated catechins, sum of EGCg, GCg and ECg; Non-gallated catechins, sum of GC, EGC, C and EC; *trans*-catechins, sum of GC, C and GCg; *cis*-catechins, sum of EGC, EC, EGCg and ECg. The direction and magnitude of correlation between variables was quantified by the correlation coefficient r. Two-tailed *p* value: one asterisk, *p* < 0.05; two asterisks, *p* < 0.01.

## 3. Experimental

### 3.1. Tea Materials and Chemicals

The green tea samples were obtained from a tea garden in the province of Boseong (34°42' N, 127°04' E), in Korea. Green teas were examined in this study by using four different leaf-age groups, and were labeled using the corresponding Korean names for each type as Woojeon (very young leaf or first flush leaf), Sejak (second flush leaf), Joongjak (third flush leaf), and Daejak (old leaf or fourth flush leaf). The tea leaves were picked from the end of April to the end of May, which was commonly regarded as plucking period for the loose tea in Korea. The tea leaves were collected from the same tea garden at intervals of one week or longer according to the weather condition and were immediately processed into commercial steamed green tea. (+)-catechin, (−)-epicatechin, (+)-catechin gallate, (−)-epicatechin gallate, (+)-gallocatechin, (−)-epigallocatechin, (+)-gallocatechin gallate, (−)-epigallocatechin gallate, caffeine, theanine, (±)-6-Hydroxy-2,5,7,8-tetramethylchromane-2-carboxylic acid (Trolox), 2,2'-Azinobis (3-ethylbenzothiazoline-6-sulfonic acid) diammonium salt (ABTS), 1,1-diphenyl-2-picrylhydrazyl (DPPH), 2,4,6-tripyridyl-s-triazine (TPTZ), ferric chloride hexahydrate, and all other chemicals used were of analytical grade and were purchased from Sigma Aldrich (St. Louis, MO, USA)

### 3.2. Green Tea Extracts and Standard Preparation

Green tea extracts were prepared by infusing green tea leaves in 100 volumes (v/w) of 50% aqueous methanol for 12 h at 25 °C in a shaking incubator. Catechins and Trolox standards for evaluation of antioxidant activity were dissolved in 20% aqueous methanol and methanol, respectively, at a concentration of 1 mM and stored at −20 *°*C. After that, stock solutions of each standard and the green tea extracts were diluted within the linear range of the assays.

### 3.3. Measurement of Theanine, Alkaloids and Catechins Contents

The contents of theanine, theobromine, caffeine and catechins in the green tea extracts were analyzed according to the modified method described by *Hu et al.* [39]. Prepared sample analyzed by HPLC (JASCO Co., Tokyo, Japan) using a XTerra RP18 column (3.5 μm, 4.6 × 150 mm, Waters, Milford, MA, USA) at 40 *°*C and multi-wavelength detector (MD-2010 Plus) was set at 210 nm. The mobile phase was composed of two solution A (0.2% orthophosphoric acid) and solution B (methanol) and eluted with a linear gradient elution of 0 min, 82% A; 15 min, 40% at a flow rate of 1.0 mL/min. Theanine, alkaloids and catechins contents were calculated by comparing with an external standard. The calibration curves were constructed by plotting concentrations *versus* peak area and showed good linearity, as described in Table 4.

**Table 4:** Calibration data for external standards using the HPLC.

Standard	r^2^	Linear Range (μg/mL)
Theanine	0.9999	18.75 *–*187.50
Theobromine	0.9999	3.75 *–*37.50
Caffeine	0.9999	7.50 *–*75.00
Gallic acid	0.9999	7.50 *–*75.00
Gallocatechin	0.9999	7.50 *–*75.00
Epigallocatechin	1.0000	7.50 *–*75.00
Catechin	1.0000	7.50–75.00
Epicatechin	0.9999	7.50 *–*75.00
Epigallocatechin gallate	0.9993	18.75 *–*187.50
Gallocatechin gallate	0.9979	18.75 *–*187.50
Epicatechin gallate	0.9979	18.75 *–*187.50
Catechin gallate	0.9962	18.75 *–*187.50

### 3.4. Measurement of Antioxidant Activity

#### 3.4.1. ABTS Assay

The ABTS assay was determined according to Thaipong *et al.* with some modifications [40]. The stock solutions included 7.4 mM ABTS and 2.6 mM potassium persulfate. ABTS radical cation was prepared by mixing the two stock solutions in equal quantities and allowed to stand for 16 h at room temperature in the dark. And then, ABTS radical solution was adjusted with phosphate buffered saline (pH 7.4) to an absorbance of 0.7*–*0.8 at 734 nm. For this assay, 190 μL of ABTS radical solution was mixed with 10 μL of each test sample solution in 96-well plates. After 60 min, the decrease of absorbance was measured at 734 nm using a microplate reader. The standard curve was linear between 0.025 and 0.9 mM Trolox. Results were expressed in mM of Trolox per g of green tea. Additional dilution was needed if the ABTS value measured was over the linear range of the standard curve.

#### 3.4.2. FRAP Assay

The FRAP assay was determined according to Benzie and Strain with some modifications [41]. FRAP reagent consist of 10 mM TPTZ in 40 mM HCl, 20 mM ferric chloride and 300 mM acetate buffer (pH 3.6) in the ratio of 1:1:10 (v/v/v). For this assay, 300 μL of FRAP reagent (at 37 °C) was mixed with 10 μL of each test sample solution in 96-well plates. After 10 min, the absorbance was measured at 593 nm using a microplate reader. The standard curve was linear between 0.003125 and 1 mM Trolox. Results were expressed in mM of Trolox per g of green tea. Additional dilution was needed if the FRAP value measured was over the linear range of the standard curve.

#### 3.4.3. DPPH Assay

The DPPH assay was determined according to Brand-Williams *et al.* with some modifications [42]. 190 μL of 0.12 mM DPPH solution was mixed with 10 μL of each test sample solution in 96-well plates. After 30 min, the decrease of absorbance was measured at 517 nm using a microplate reader. The standard curve was linear between 0.025 and 1 mM Trolox. Results were expressed in mM of Trolox per g of green tea. Additional dilution was needed if the DPPH value measured was over the linear range of the standard curve.

### 3.5. Statistical Analysis

All experimental data were expressed as mean and standard deviation and analyzed by one-way ANOVA followed by Tukey’s *post hoc* test at *p* < 0.05 using the SPSS program (SPSS Inc., Chicago, IL, USA). Additionally, the most important factors contributing to the antioxidant activity of green tea extracts were determined using Pearson’s correlation coefficients.

## 4. Conclusions

The chemical compositions and antioxidant activities of green tea with different plucking periods were determined and the resultant data were subjected to correlation analysis to find the important factors contributing to the antioxidant activity. Additionally, we have re-examined the apparent relationship between individual catechins and antioxidant activity using individual catechin standards with Trolox to verify it. The results show that the antioxidant activities of green teas are dependent on their chemical composition and especially on the presence of *cis*-catechins, found in high contents in relatively old leaves. As such, these findings suggest that the leaf age-dependent changes in catechin composition have the potential to increase the antioxidant activity of green tea and they may possibly be controlled by the agronomic conditions under which the tea is grown. It is expected that the results obtained in the present study will provide a broader understanding of the bioactive compounds of green tea, and enable more informed decisions to be made regarding the plucking polices of tea leaves.
